# Development and External Validation of a Machine Learning Tool to Rule Out COVID-19 Among Adults in the Emergency Department Using Routine Blood Tests: A Large, Multicenter, Real-World Study

**DOI:** 10.2196/24048

**Published:** 2020-12-02

**Authors:** Timothy B Plante, Aaron M Blau, Adrian N Berg, Aaron S Weinberg, Ik C Jun, Victor F Tapson, Tanya S Kanigan, Artur B Adib

**Affiliations:** 1 Larner College of Medicine at the University of Vermont Colchester, VT United States; 2 University of Vermont Medical Center Burlington, VT United States; 3 Larner College of Medicine at the University of Vermont Burlington, VT United States; 4 Biocogniv Inc South Burlington, VT United States; 5 Cedars-Sinai Medical Center Los Angeles, CA United States

**Keywords:** COVID-19, SARS-CoV-2, machine learning, artificial intelligence, electronic medical records, laboratory results, development, validation, testing, model, emergency department

## Abstract

**Background:**

Conventional diagnosis of COVID-19 with reverse transcription polymerase chain reaction (RT-PCR) testing (hereafter, PCR) is associated with prolonged time to diagnosis and significant costs to run the test. The SARS-CoV-2 virus might lead to characteristic patterns in the results of widely available, routine blood tests that could be identified with machine learning methodologies. Machine learning modalities integrating findings from these common laboratory test results might accelerate ruling out COVID-19 in emergency department patients.

**Objective:**

We sought to develop (ie, train and internally validate with cross-validation techniques) and externally validate a machine learning model to rule out COVID 19 using only routine blood tests among adults in emergency departments.

**Methods:**

Using clinical data from emergency departments (EDs) from 66 US hospitals before the pandemic (before the end of December 2019) or during the pandemic (March-July 2020), we included patients aged ≥20 years in the study time frame. We excluded those with missing laboratory results. Model training used 2183 PCR-confirmed cases from 43 hospitals during the pandemic; negative controls were 10,000 prepandemic patients from the same hospitals. External validation used 23 hospitals with 1020 PCR-confirmed cases and 171,734 prepandemic negative controls. The main outcome was COVID 19 status predicted using same-day routine laboratory results. Model performance was assessed with area under the receiver operating characteristic (AUROC) curve as well as sensitivity, specificity, and negative predictive value (NPV).

**Results:**

Of 192,779 patients included in the training, external validation, and sensitivity data sets (median age decile 50 [IQR 30-60] years, 40.5% male [78,249/192,779]), AUROC for training and external validation was 0.91 (95% CI 0.90-0.92). Using a risk score cutoff of 1.0 (out of 100) in the external validation data set, the model achieved sensitivity of 95.9% and specificity of 41.7%; with a cutoff of 2.0, sensitivity was 92.6% and specificity was 59.9%. At the cutoff of 2.0, the NPVs at a prevalence of 1%, 10%, and 20% were 99.9%, 98.6%, and 97%, respectively.

**Conclusions:**

A machine learning model developed with multicenter clinical data integrating commonly collected ED laboratory data demonstrated high rule-out accuracy for COVID-19 status, and might inform selective use of PCR-based testing.

## Introduction

SARS-CoV-2 is the cause of COVID-19, which continues to spread in an uncontrolled manner across the United States [1]. COVID-19 management includes patient isolation and supportive care [2]. This strategy requires expeditious COVID-19 diagnosis, but components required for the reverse transcription polymerase chain reaction (RT-PCR; hereafter, PCR) assay have been reported to be in short supply in some locations during the pandemic, leading to delays in results [3]. In the absence of a widely available PCR test with rapid turnaround, there is an urgent need to identify alternative means for stratifying risk of patients seeking care during the COVID-19 pandemic.

Risk assessment models might identify those at low risk of active COVID-19 using available data from the clinical encounter [4,5]. In contrast to traditional model-building techniques, machine learning technologies consider complex linear and nonlinear associations between independent variables and identify characteristic patterns of commonly collected data among patients with COVID-19 [6]. A test with high sensitivity and diagnostic yield (ie, fraction of patients ruled out) could be used in a manner analogous to other rule-out tests, such as D-dimer for pulmonary embolism [7].

Using emergency department (ED) patient encounters from a well-established multicenter clinical database, we sought to describe the development of a machine learning model for ruling out COVID-19 using only routinely collected laboratory tests. Furthermore, we aimed to assess the area under the receiver operating characteristic (AUROC) curve of a machine learning model’s concordance with both COVID-19 PCR test results (for positives) and prepandemic patients (for negatives). We hypothesized that such a machine learning model would enable the ruling out of the disease with sensitivity >90% and diagnostic yield >50%.

## Methods

### Study Design and Setting

This analysis and its reporting is compliant with the Standards for Reporting Diagnostic Accuracy Studies (STARD) statement [8]. This cross-sectional study was performed using 3 data sets of deidentified, patient-level electronic medical records of adult patients in an ED. The Premier Healthcare Database (PHD) is a large database of 1041 US hospitals from all 9 US geographic regions defined by the US Census [9]. At time of writing, 155 hospitals contribute SARS-CoV-2 RNA testing results to the PHD. We separately obtained data from Cedars-Sinai Medical Center (CSMC), an 886-bed academic medical center in Los Angeles, CA, and the Beth Israel Deaconess Medical Center (BIDMC), a 673-bed academic medical center in Boston, MA. An inclusion flow diagram and descriptions of these data sets are provided in Section A of Multimedia Appendix 1.

### Prepandemic and Pandemic Time Frames

Two time frames were used, defined by the date of ED visit: prepandemic (before January 2020) and pandemic (March 2020 through July 2020). January 2020 and February 2020 were not included due to the lack of widespread monitoring or diagnostic tests for COVID-19 in the United States during this time frame, even though SARS-CoV-2 community transmission was present in the United States during this time [10]. Clinical encounter data from the PHD were available for the prepandemic (January 2019-December 2019) and pandemic (March 2020 through July 2020) time frames. CSMC data were available for patients with COVID‑19 during the pandemic time frame only (March-April 2020). BIDMC data were available across an extended prepandemic time frame (2008-2019) only for patients who were admitted through the ED.

### Selection of Participants

Eligible patient encounters (hereafter, patients) were adults aged ≥20 years in an ED at an included center during one of the prepandemic or pandemic time frames. Patients were excluded if they were missing a laboratory result included in the model on the day of presentation to the ED or if any of their laboratory results were reported with inappropriate units or incorrect specimen type. Patients were defined as PCR-positive for COVID-19 (hereafter, PCR-positive) if they had a positive SARS-CoV-2 RNA test on the day of presentation to the ED. We chose PCR rather than antigen positivity to define the cases as PCR is commonly used as the reference standard in COVID-19 diagnosis [11,12].

### Training Population and Definition of COVID-19 Cases and Controls

Training occurred in the PHD database only. The PHD training and external validation sets were split by hospital, and only hospitals that reported COVID positives as well as the blood tests required for the model were included in the analysis (64 total). Of these, 43 hospitals were randomly assigned to the training set, and 21 to the external validation set (hereafter, PHD holdout). Cases came from the pandemic time frame, and any patients in this time frame without a positive PCR test were excluded. Contemporary COVID-19 PCR assays have elevated false negative rates, which could lead to mislabeled data and hence to degraded model performance [13]. Due to this, prepandemic controls randomly selected from the 43 PHD hospitals in the training set were used in place of PCR-negative patients during the pandemic.

### External Validation Populations

The external validation data set used 3 data sources: 952 PCR-positives and 154,341 prepandemic visits from the 21 hospitals in the PHD holdout set; 68 PCR-positive patients from CSMC; and 17,393 prepandemic (2008-2019) patient encounters from BIDMC. Patients in the pandemic time frame without a positive PCR test were excluded. All prepandemic patients were treated as negatives when evaluating the performance of the model in predicting COVID-19 status. The prepandemic patients from the PHD holdout were chosen so as to match the top 20 most frequent primary diagnoses given to patients without COVID-19 during the pandemic, as coded by Clinical Classifications Software Refined (CCSR) codes (listed in Section B of Multimedia Appendix 1).

### Sensitivity Analysis Population

To evaluate how the model generalizes to pandemic time frame patients only, we performed a sensitivity analysis using patients presenting to the ED in the 21 centers from the PHD holdout with any SARS-CoV-2 PCR result available on day of presentation. This differed from the other analyses as negatives were from the same time frame as the positives. This resulted in a total of 952 PCR-positive patients and 6890 PCR-negative patients in the pandemic period (March-July 2020).

### Subgroup Analyses

The AUROC was tabulated by decile of age, sex, race, admission or discharge status, and intensive care unit (ICU) admission status in the external validation data set. The distribution of risk scores was also visualized for all studied cohorts through box plots. For PCR-positives, this included positives from CSMC, and PHD visits that had a single positive PCR result as well as visits that had a negative result before a positive result (both on the day of presentation). For PCR negatives during the pandemic, this included patients with both single- and double-PCR results on the day of presentation. For prepandemic encounters, the scores for all eligible BIDMC patients were considered, as well as those from the PHD holdout that matched the top 20 CCSR (non–COVID-19) codes observed during the pandemic.

### Model Development (Training and Internal Validation With Cross-Tabulation Techniques)

The model was intended to estimate COVID-19 status on the day of presentation to an ED using common laboratory tests collected that day. Model training began with 29 routinely measured features (ie, potential or included model covariates) comprising the comprehensive metabolic panel and the complete blood count with differential. Recursive feature elimination with cross-validation (RFECV) was performed to arrive at the final 15 features [14]. We used the gradient boosting model as implemented in XGBoost [15] for all results. No hyperparameter optimization was performed and default parameters were used. Performance on the training set was evaluated through stratified 5-fold cross-validation. Performance in the external validation and sensitivity analysis data sets was obtained after training the model on the entire training set.

### Statistical Analysis

Baseline demographics, ED disposition, and included laboratory features from the training, external validation, and sensitivity analysis data sets were tabulated by COVID-19 status. Visualization of the distribution of features used box plots, ordered by feature importance (compare with list values in Section C of Multimedia Appendix 1). Model discrimination was visualized with receiver operating characteristic (ROC) curves and estimation of the AUROC. AUROC 95% CIs were estimated with bootstrapping. Hosmer-Lemeshow criteria were used to describe performance of discrimination [16]. These criteria considered an AUROC value of 0.5 as no discrimination, 0.5 to <0.7 as poor discrimination, 0.7 to <0.8 as acceptable discrimination, 0.8 to <0.9 as excellent discrimination, and ≥0.9 as outstanding discrimination. Sensitivity, specificity, and negative predictive value (NPV) were defined using conventional definitions. Diagnostic yield was defined as the percentage of patients with a risk score below a given cutoff. All analyses were prespecified. The sample size of this analysis was driven by data availability in this multicenter database.

Analyses were performed in Python (Version 3.7.5; Python Software Foundation) using the XGBoost package (Version 0.82) [17] and the Scikit-Learn library (Version 0.21.3) [18]. The use of deidentified databases as described here met the non–human subjects research by the University of Vermont’s Institutional Review Board criteria.

## Results

### Demographics and Proportion of PCR-Positive Patients in Training Data Set, External Validation Data Set, and Sensitivity Analysis Data Set

The training data set included 12,183 ED visits at 43 centers from the PHD, of which 2183 results were PCR-positive. The validation data set included 172,754 ED visits from 23 centers (21 from the PHD, as well as the independently collected data from CSMC and BIDMC), of which 1020 results were PCR-positive. The sensitivity analysis data set included 7842 records from 21 centers in the PHD holdout group. Patient demographics and visit characteristics are summarized in Table 1.

A total of 192,779 eligible patients were included in the study; the median age decile was 50 (IQR 30-60) years and 40.5% (78,249/192,779) were male. In the training, external validation, and sensitivity analysis data sets, the median age deciles were 50 (IQR 30-70) years, 50 (IQR 30-60) years, and 50 (IQR 40-70) years, respectively. Males represented 42.9%, 40.1%, and 47.4% of patients in the data sets, respectively.

**Table 1:** Demographics of patients and encounter details, by COVID-19 status^a^.

Demographics		Training (N=12,183)		External validation (N=172,754)			Sensitivity analysis (N=7842)		
		Negative (n=10,000)	Positive (n=2183)		Negative (n=171,734)	Positive (n=1020)		Negative (n=6890)	Positive (n=952)
**Age (years), n (%)**									
	20 to <30	1392 (14)	198 (9)		27,952 (16)	71 (7)		709 (10)	70 (7)
	30 to <40	1481 (15)	304 (14)		29,187 (17)	127 (12)		882 (13)	119 (12)
	40 to <50	1398 (14)	413 (19)		27,764 (16)	214 (21)		896 (13)	205 (22)
	50 to <60	1649 (16)	400 (18)		28,896 (17)	217 (21)		1172 (17)	208 (22)
	60 to <70	1512 (15)	367 (17)		23,771 (14)	180 (18)		1200 (17)	163 (17)
	70 to <80	1322 (13)	264 (12)		18,460 (11)	121 (12)		1063 (15)	108 (11)
	≥80	1246 (12)	237 (11)		15,704 (9)	90 (9)		968 (14)	79 (8)
**Gender, n (%)**									
	Female	5876 (59)	1079 (49)		102,942 (60)	502 (49)		3650 (53)	477 (50)
	Male	4122 (41)	1104 (51)		68,790 (40)	518 (51)		3240 (47)	475 (50)
	Unknown	2 (0)	0 (0)		2 (0)	0 (0)		0 (0)	0 (0)
**Race, n (%)**									
	Black	1791 (18)	397 (18)		28,874 (17)	212 (21)		1230 (18)	201 (21)
	Other	904 (9)	976 (45)		23,222 (14)	453 (44)		772 (11)	448 (47)
	Unknown	450 (4)	102 (5)		12,284 (7)	48 (5)		368 (5)	36 (4)
	White	6855 (69)	708 (32)		107,354 (63)	307 (30)		4520 (66)	267 (28)
**Census division^b^, n (%)**									
	East North Central	2065 (21)	280 (13)		16,184 (9)	108 (11)		1103 (16)	108 (11)
	East South Central	0 (0)	0 (0)		3549 (2)	50 (5)		138 (2)	50 (5)
	Middle Atlantic	782 (8)	294 (13)		18,776 (11)	92 (9)		1356 (20)	92 (10)
	New England	493 (5)	1 (0)		31,624 (18)	1 (0)		1 (0)	1 (0)
	Pacific	106 (1)	32 (1)		3617 (2)	69 (7)		34 (0)	1 (0)
	South Atlantic	3116 (31)	1192 (55)		70,463 (41)	613 (60)		2790 (40)	613 (64)
	West North Central	633 (6)	39 (2)		0 (0)	0 (0)		0 (0)	0 (0)
	West South Central	2805 (28)	345 (16)		27,521 (16)	87 (9)		1468 (21)	87 (9)
**Rural or urban^b^, n (%)**									
	Rural	583 (6)	21 (1)		3617 (2)	1 (0)		34 (0)	1 (0)
	Urban	9417 (94)	2162 (99)		168,117 (98)	1019 (100)		6856 (100)	951 (100)
**Disposition, n (%)**									
	Discharge from emergency department	7487 (75)	1175 (54)		132,195 (77)	522 (51)		4072 (59)	522 (55)
	Non–intensive care unit admission	2068 (21)	805 (37)		29,793 (17)	379 (37)		2375 (34)	335 (35)
	Intensive care unit admission	445 (4)	203 (9)		9746 (6)	119 (12)		443 (6)	95 (10)

^a^For the training data set: COVID-19 positivity was defined as a positive COVID-19 reverse-transcription polymerase chain reaction (hereafter, PCR) test on the day of presentation to the emergency department among patients in the pandemic time frame (March 2020 through July 2020) in the Premier Healthcare Database (PHD) database among a random selection of 43 of the 64 PHD hospitals reporting PCR positives. COVID-19 negativity was defined as a selection of 10,000 patients in the prepandemic time frame (January through December 2019) in the PHD database from the same 43 hospitals as the patients with COVID-19. For the external validation data set: COVID-19 positivity was defined the same as for the training data set for the PHD data set but also included 952 PCR-positives from the 21 hospitals in the PHD holdout set. Additionally, it included 68 patients with PCR-confirmed COVID-19 from Cedar Sinai Medical Center from March and April 2020. COVID-19 negativity in the external validation set was defined using 154,341 prepandemic visits from the 21 hospitals in the PHD holdout set (January through December 2019) in which primary diagnoses were among the 20 most frequent primary diagnoses given to patients negative for COVID-19 during the pandemic, using Clinical Classification Software Refined codes. It also included 17,393 prepandemic (2008-2019) patient encounters from Beth Israel Deaconess Medical Center. For the sensitivity data set: COVID-19 positivity included the same 952 PCR-positives from the 21 hospitals in the external validation data set. COVID-19 negativity was defined as visits with at least 1 PCR-negative but no PCR-positive result on the day of presentation, and included all 6890 patients with such results from the same 21 hospitals as the positives.

^b^Census division was defined using US Census classification [19]. Rural areas are considered territory outside of the US Census Bureau’s definition of urban [20]. These geographic descriptions pertain to the hospital, not the patient’s permanent residence.

### Selected Features Included in the Model and Individual Feature Performance

The RFECV method led to the final set of 15 features listed in Table S1 in Multimedia Appendix 1. The distributions of these features in the training data set, stratified by COVID-19–positive and COVID-19–negative status and ordered by importance to the model, are shown in Figure S1 in Multimedia Appendix 1. The features with the largest calculated importance were eosinophils, calcium, and aspartate aminotransferase. Summary statistics of these features in the training, external validation, and sensitivity analysis data sets, stratified by COVID-19 status, appear in Table S1 in Multimedia Appendix 1.

### Performance of Individual Features and Model Performance in the Training Data Set

**Figure 1:**
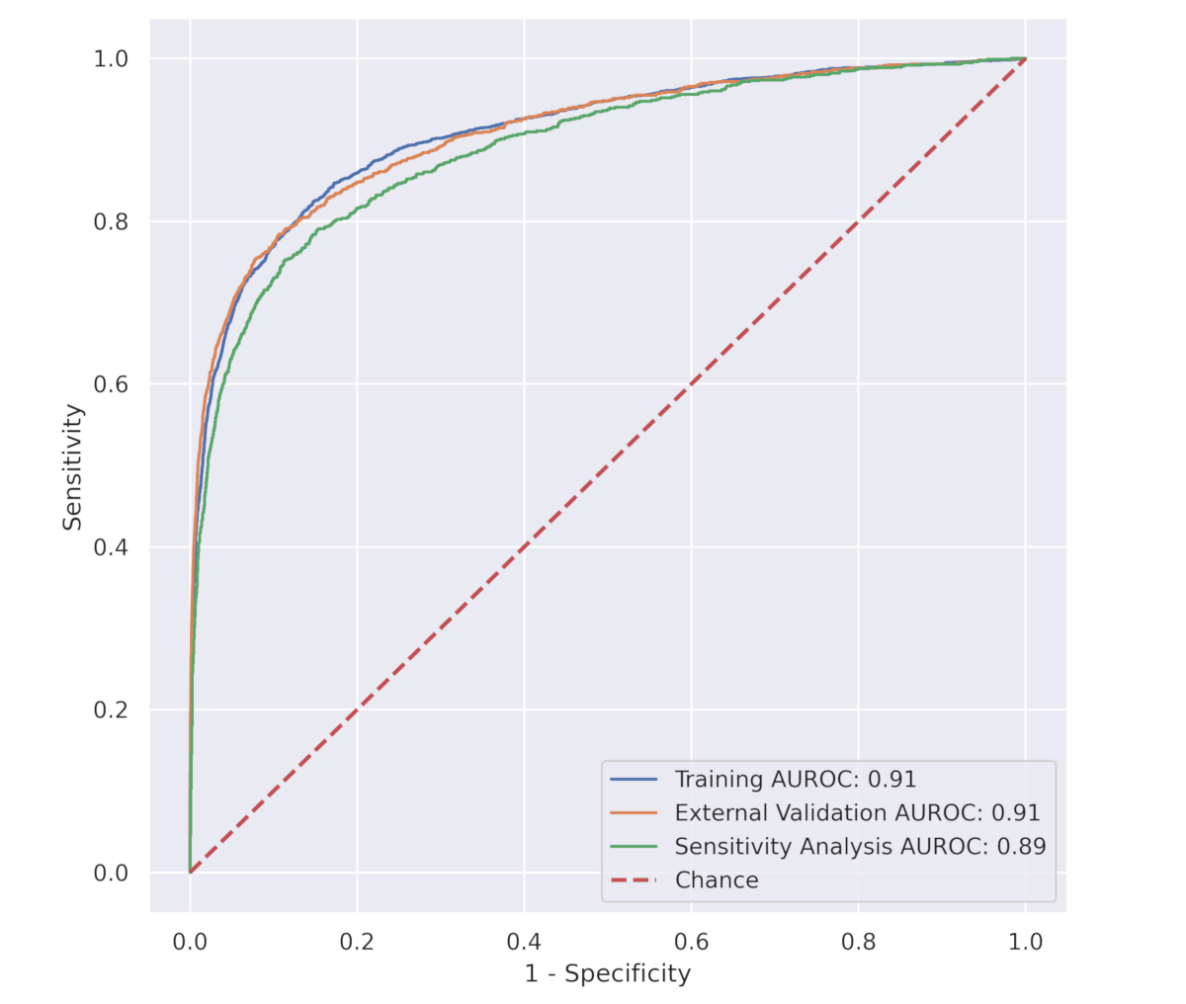
Discrimination as assessed by ROC curves for training, external validation, and sensitivity analysis data sets. ROC curves for the 3 different data sets: training (blue), external validation (orange), and sensitivity analysis (green). The training curve was obtained through 5-fold cross-validation, where positive controls are PCR-confirmed cases during the pandemic (N=2183) and negative controls are prepandemic patients (N=10,000) from 43 hospitals in the PHD. The training AUROC was 0.91 (95% CI 0.90-0.92). The external validation curve was performed in the external validation data set after training the model on the training data set. External validation positives are PCR-confirmed cases from Cedars-Sinai Medical Center (N=68) and from the PHD holdout set (N=952) comprising 21 hospitals. External validation negatives are prepandemic (2019) patients, from the same 21 PHD hospitals, that match the top 20 primary non–COVID-19 diagnoses in 2020 (N=154,341), as well as all eligible prepandemic (2008-2019) Beth Israel Deaconess Medical Center patients (N=17,393). The AUROC in the external validation data set was 0.91 (95% CI 0.90-0.92). The sensitivity analysis curve demonstrates the effect of using prepandemic patients as negative controls compared to using PCR-negatives from 2020. In this data set, both positives (N=952) and negatives (N=6890) were PCR-confirmed patients from the PHD holdout set (21 hospitals), and no prepandemic data was included. The AUROC in the sensitivity analysis set was 0.89 (95% CI 0.88-0.90). AUROC: area under the receiver operating characteristic curve; PCR: polymerase chain reaction; PHD: Premier Healthcare Database; ROC: receiver operating characteristic.

The AUROC for each individual feature in the training data set is shown in Figure S2 in Multimedia Appendix 1. The highest AUROCs were observed for eosinophils, calcium, and aspartate aminotransferase (0.70-0.80). The final model’s AUROC in the training data set was 0.91 (95% CI 0.90-0.92; Figure 1).

### Model Performance in the External Validation Data Set

The model’s AUROC in the external validation data set was 0.91 (95% CI 0.90-0.92), as shown in Figure 1. This corresponds to an outstanding discrimination per the Hosmer-Lemeshow criteria [16]. Sensitivity and specificity were 95.9 and 41.7 at a score cutoff of 1, 92.6 and 60.0 at a score of 2, 85.5 and 78.5 at a cutoff of 5, and 79.4 and 87.6 at a cutoff of 10, respectively (Table 2).

With a COVID-19 population prevalence of 1%, each of these cutoffs had an NPV >99%; at 10% prevalence, each was >97%, and at a prevalence of 20%, each was >94%. The diagnostic yield ranged from 34% (20% prevalence, score cutoff of 1) to 87% (1% prevalence, score cutoff of 10).

**Table 2:** Clinical performance metrics for the model in the external validation data set for various score cutoffs and COVID-19 pretest prevalence^a^.

Score cutoff	Sensitivity	Specificity	Likelihood ratio^b^	Prevalence of 1%		Prevalence of 10%		Prevalence of 20%	
				NPV^c^, %	Yield^d^, %	NPV, %	Yield, %	NPV, %	Yield, %
1	95.9	41.7	0.099	99.9	41.3	98.9	38.0	97.6	34.2
2	92.6	60.0	0.124	99.9	59.4	98.6	54.7	97.0	49.5
5	85.5	78.5	0.185	99.8	77.8	98.0	72.1	95.6	65.7
10	79.4	87.6	0.235	99.8	86.9	97.4	80.9	94.4	74.2

^a^The maximum score was 100; a higher score indicates higher model prediction of COVID-19 positivity.

^b^The likelihood ratio uses the equation for negative tests.

^c^NPV: negative predictive value.

^d^Yield refers to diagnostic yield, which is the percentage of patients that can be ruled out (ie, those with a score below the cutoff).

### Sensitivity Analysis and Subgroup Analyses

**Figure 2:**
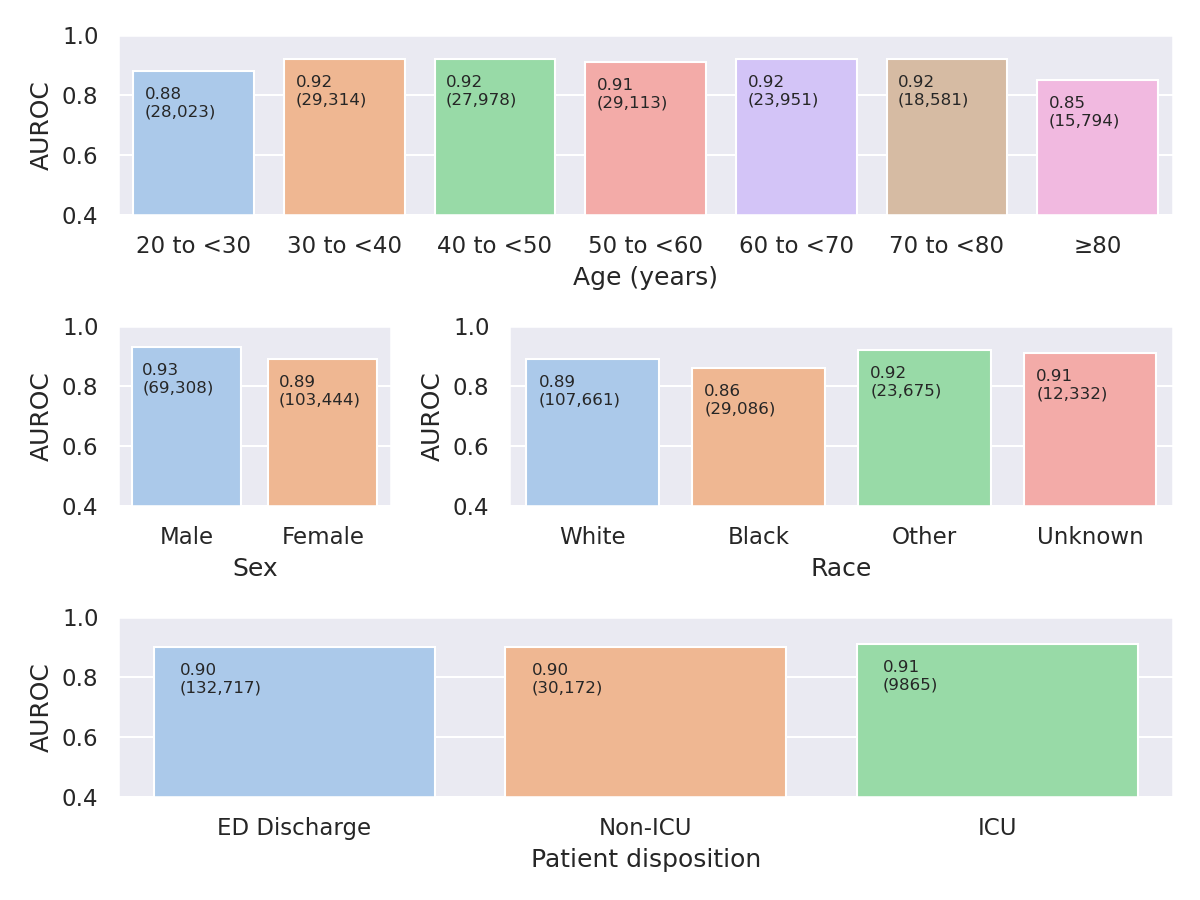
Discrimination as assessed by AUROC curve in age, sex, race, and ED disposition subgroups in the external validation data set. Non-ICU patients were admitted to the hospital but not to an ICU. Distribution of AUROC curves per demographic, as well as per patient disposition type (ED discharge, non-ICU, and ICU) in the external validation data set. Top numbers are AUROC curves, bottom numbers in parentheses are the number of patients. AUROC: area under the receiver operating characteristic; ED: emergency department; ICU: intensive care unit.

Figure 1 depicts the ROC curve in the sensitivity analysis data set, which contains only year 2020 patients with PCR-confirmed positive and negative results (ie, no historical negatives). The AUROC was 0.89 (95% CI 0.88-0.90). In Figure 2, the AUROC is presented for various demographic cohorts as well as patient disposition (ED discharge, non-ICU, and ICU) in the external validation data set. AUROCs ranged from 0.86 to 0.93. AUROC by these subgroups was similar in the sensitivity analysis data set, which appears as Figure S3 in the Multimedia Appendix 1.

### Distribution of Risk Scores in Selected Subgroups

Lastly, an extensive distribution of risk scores for various subgroups is shown in Figure S4 in Multimedia Appendix 1, including prepandemic patients whose primary diagnoses were among the top 20 primary diagnoses among patients without COVID-19 in 2020. From visual inspection, it can be seen that high scores track PCR-positive patients consistently across all cohorts.

## Discussion

### Principal Results

A development and external validation study of a machine learning model for COVID-19 status using laboratory tests routinely collected in adult ED patients found high discrimination across age, race, sex, and disease severity subgroups. This model had high diagnostic yield at low score cutoffs in a screening population with a disease prevalence of <10%. Such a model could rapidly identify those at low risk for COVID-19 in a “rule-out” method, and might reduce the need for PCR testing in such patients.

### Comparison With Prior Work

Prior literature has described the application of machine learning techniques to commonly collected laboratory data for estimation of missing laboratory analytes. For example, an analysis by Waljee and colleagues [21] leveraged machine learning techniques for imputation of missing laboratory data in cohorts of patients with cirrhosis and inflammatory bowel disease at a single institution. In comparison to other common imputation techniques described in this manuscript, the machine learning technique introduced the least imputation error for these laboratory data. Luo and colleagues [22] used similar methods to estimate ferritin from a single medical center, and found that the machine learning technique outperformed traditional imputation methods. These serve as strong evidence of the potential use of machine learning for use in estimation of laboratory data. However, outside of imputation of missing values from research databases, the clinical utility for such techniques was unclear prior to the COVID-19 pandemic.

During this pandemic, there is an urgent need to rapidly identify patients with the disease to inform supportive clinical care. Prior work has attempted to integrate combinations of clinical data points in diagnostic models, though only a few are currently published in peer-reviewed literature [23]. The selection of the specific data points to integrate into machine learning models for COVID-19 diagnosis has implications on integration into existing clinical delivery. In contrast with the results here, which only included components of the routinely collected complete blood count with differential and complete metabolic panel laboratory tests, others have integrated nonlaboratory features. Sun and colleagues [24] reported 3 models including demographics, radiological data, and symptomatology, and obtained AUCs ranging from 0.65 to 0.88 for these models. Symptomatology was not obtained with structured, validated questionnaires and the ability to capture these symptoms in a reproducible manner might be difficult outside of a research setting. Further, modern medical records cannot integrate such symptoms into automated risk scores as they are not documented in a structured way.

Structured data obtained routinely in clinical examinations are the simplest to integrate, and might have the least variability between institutions. These include vital signs, demographics, laboratory findings, and radiological images. There are few studies describing the use of such data for the diagnosis of COVID-19. One study found a machine learning method had an accuracy of 87% for distinguishing between COVID-19 from pneumonia or no relevant findings using chest radiographs [25]. A different model developed from chest computed tomography images reported an AUROC of 0.994 when distinguishing between COVID-19 and atypical or viral pneumonia [26]. However, national organizations recommend against the use of radiological imaging for diagnosis of COVID-19, in part because of the added risk of spreading infection through additional visitation to radiology suites [27]. These models are unlikely to be readily deployed because of the challenges of performing elective radiological tests during this pandemic.

An additional consideration in the development of machine learning models is the inclusion of an adequate sample size for model training [28-30]. Other studies have investigated the role of laboratory data with or without other nonradiological structured clinical data or demographics for the diagnosis of COVID-19 using machine learning techniques. For example, Wu and colleagues [31] reported a C-index of 0.99 but included only 108 patients (12 COVID-19–positive) in their training. Similarly, individual efforts led by Batista, Brinati, and Soares [32-34] describe machine learning models trained on 234, 279, and 599 patients, respectively. These studies are also limited in the small number of centers from which patients were enrolled, and lack of diversity in their patient populations.

### Advancement of Scientific Knowledge

The present analysis advances science in several key ways. First, we describe a machine learning model developed in a diverse patient population with routine laboratory data from multiple clinical centers across the United States [35]. Second, the model incorporates common laboratory tests that are widely available with rapid turnaround time. As the machine learning model can be performed essentially instantaneously, the primary time limitation is related to phlebotomy and specimen processing. There is a well-known bottleneck in completing conventional COVID-19 PCR assays; a commercial laboratory recently reported a 7-day reporting lag [36]. Third, the present model could identify those at lowest risk for COVID-19 to inform a “rule-out” method for screening. Those with intermediate or greater risk for COVID-19 could be further assessed with COVID-19 PCR testing, if indicated. Depending on the selected score cutoff and population prevalence, such an approach could rule out 34% to 87% of ED patients requiring conventional COVID‑19 PCR testing (see Yield, Table 2). The specific score cutoff for rule out of COVID-19 with this model can be customized based upon what an institution considers to be an “acceptable” target NPV. However, the diagnostic yield will change based upon the screening population prevalence of COVID-19, and the diagnostic yield will be inversely related to the screening population prevalence of COVID-19. For example, assume that an institution determines that an acceptable NPV for this model is 97.5%. If this institution’s screening population has a 20% prevalence of COVID-19, the threshold score cutoff would be set at 1, and the diagnostic yield (ie, the percentage of patients ruled out for COVID-19 at a score cutoff of 1) would be 34.2% (Table 2). However, at a prevalence of 10%, the score cutoff threshold would be 10, and the diagnostic yield would be 80.9%. The efficiency of diagnostic yield with this model is higher at lower prevalence. Finally, the sensitivity of the present model at a score cutoff of 1, 2, and 5 (95.9, 92.6, and 85.5, respectively) was similar to COVID-19 antigen assays (66.1-86.3) and sputum and saliva PCR assays (62.3-97.2) [11]. The comparatively similar sensitivities between the model and these existing assays supports the clinical utility of machine learning models as future diagnostic tools.

### Weaknesses and Strengths

This study has weaknesses. Although the choice of prepandemic controls partially circumvents the issue of false negatives in PCR testing by ensuring the negatives that the model is trained on are true negatives, it does not ensure that the positives encompass the full spectrum of true positives, since those are sometimes missed by PCR due to changes in viral load as a function of disease progression [37,38]. Additionally, the use of controls from a different time period could introduce a bias of its own, such as different demographics or non–COVID-19 morbidities. However, the sensitivity analysis used COVID-19 positives and negatives from the pandemic time frame, and the performance of the model was reassuringly similar to the performance in the external validation. The performance in demographic, clinical diagnosis, and ED disposition subgroups was also similar to the external validation. Laboratory data were performed locally at each hospital, rather than centrally. The model requires all components of the laboratory data to be included. This study only included patients who visited an ED. Although it is likely that some of the patients in this study were asymptomatic or presymptomatic and were found to have COVID-19 as part of routine admission, we were unable to determine the indications for screening and therefore are unable to determine the performance of this model in asymptomatic and presymptomatic adults. The present analysis only accounted for results from COVID-19 PCR tests and not for alternative diagnostic methods, such as antigen testing for acute infection or antibody testing to demonstrate prior infection. Finally, the research database did not include details about the specific PCR assay used in diagnosis, so we are unable to comment on performance of the model in comparison to the performance of the specific assays.

This study has strengths. This study included data from a large number of patients and hospitals, and to our knowledge is the largest application of machine learning to COVID-19. Data were derived from an electronic medical records database that is commonly used in clinical research. The patient population was geographically and racially diverse. The only features included in the model are those included in blood tests that are already routinely collected in ED encounters. Further, these tests were from multiple hospitals, suggesting that the model is robust against different specimen collection, handling practices, and instrumentation. Sensitivity analyses were performed to evaluate potential biases due to the choice of prepandemic negative controls, and no significant bias was observed across multiple dimensions. Our methods extend on established machine learning–based imputation methods for missing laboratory data [21,22], and suggest there may be clinical utility of these techniques in ruling out the disease. Finally, the external validation was a true external validation since it used data from hospitals that were not included in the training data set. This supports the resilience of the model across institutions with differing specimen handling and laboratory processing methods.

### Conclusions

A machine learning model for ruling out COVID-19 in ED patients that integrates commonly collected laboratory data had a discrimination accuracy that can be classified as excellent to outstanding [16]. Using score cutoffs of 5 and 10 points, and assuming a 10% screening population prevalence of COVID-19, 72% and 81% of patients were ruled out with this model while maintaining an NPV >97%, respectively.

## Appendix

Multimedia Appendix 1 Supplementary figures and tables.

## Abbreviations

AUC: area under the curve
AUROC: area under the receiver operating characteristic
BIDMC: Beth Israel Deaconess Medical Center
CCSR: Clinical Classifications Software Refined
CSMC: Cedars-Sinai Medical Center
ED: emergency department
ICU: intensive care unit
NPV: negative predictive value
PCR: reverse transcription polymerase chain reaction
PHD: Premier Healthcare Database
RFECV: recursive feature elimination with cross-validation
ROC: receiver operating characteristic
STARD: Standards for Reporting Diagnostic Accuracy Studies
