# Supplementary material for: Development and External Validation of a Machine Learning Tool to Rule Out COVID-19 Among Adults in the Emergency Department Using Routine Blood Tests: A Large, Multicenter, Real-World Study
Source: J Med Internet Res. 2020 Dec 2;22(12):e24048. doi: 10.2196/24048 (PMC7713695; doi:10.2196/24048)
Supplement: Multimedia Appendix 1 [file jmir_v22i12e24048_app1.docx]

# Supplemental Appendix

**Development and External Validation of a Machine Learning Tool to Rule Out COVID-19 Among Adults in the Emergency Department Using Routine Blood Tests: A Large, Multicenter, Real-World Study**

Timothy B Plante, MD MHS, Aaron Blau, MD, Adrian N Berg, BS, Aaron S Weinberg, MD MPhil, Ik C Jun MD, Victor F Tapson, MD, Tanya S Kanigan, PhD, Artur B Adib, PhD

- **Pg. 2-3:** Section A. Description of the databases and inclusion flow diagram
- **Pg. 4:** Section B. List of CCSR codes used to select controls in the PHD holdout dataset
- **Pg. 5:** Section C: Feature importance and AUROCs
- **Pg. 6:** Supplemental Table S1: Summary of Laboratory Results Grouped by Panel for Patients Included in Study- by COVID-19 status
- **Pg. 7:** Supplemental Figure S1 - Fifteen included features in the training dataset, by COVID-19 status
- **Pg. 8:** Supplemental Figure S2 - AUROCs for individual features and the overall model
- **Pg. 9:** Supplemental Figure S3 -
- **Pg.** **10**: Supplemental Figure S4 - Distribution of risk scores in the validation dataset

## Section A. Description of the Databases and Inclusion Flow Diagram

### Premier Healthcare Database (PHD)

The Premier Healthcare Database (PHD) is a large, US. hospital-based, service-level, all-payer database that contains information on inpatient and outpatient discharges, primarily from geographically diverse non-profit, non-governmental and community and teaching hospitals and health systems from rural and urban areas. Since 2000, more than 600 peer-reviewed publications have used this dataset.

From the PHD, we defined eligible visits as those where patients presented to the hospital through the emergency department (ED), had complete blood count (CBC) and a comprehensive metabolic panel (CMP) ordered on the day of presentation, and received at least 1 SARS-CoV-2 reverse transcription polymerase chain reaction (hereafter, PCR) test on that same day. Rare inconsistencies in the PCR data, such as discrepant results from the same specimen, were removed.

The eligible visits were divided into 2 mutually exclusive groups by hospital, labelled “training” (43 hospitals, or ~2/3 of total) and the “PHD holdout” (21 hospitals, or ~1/3 of total), which is included in the validation set along with Cedars-Sinai Medical Center (CSMC) and Beth Israel Deaconess Medical Center (BIDMC). Hospitals that never reported PCR-positive results were excluded from the analysis.

For the training set, patients who had at least 1 PCR-positive test result were labelled “positive” (2,183 patients). Due to the concern with high false negative rates, we did not train our model on patients with PCR-negative results. Instead, for negative controls we selected a random sample of 10,000 pre-pandemic (2019) patients who presented to the ED of the same 43 hospitals and had CBC and CMP ordered.

For the validation of the model, we used an entirely separate subset from the PHD that the model didn’t have access to during development (PHD holdout). This holdout set contains 952 PCR-positive visits and 154,341 pre-pandemic patients from those same 21 hospitals.

### Cedars-Sinai Medical Center

De-identified data were collected from patients that presented to the CSMC ED between March and April of 2020 that tested positive for SARS-CoV-2 virus by PCR within the first several hours of their ED visit (79 patients). Patients who did not have all the required laboratory results for the model (11 patients) were excluded from the analysis. This data collection was reviewed by the Cedars-Sinai institutional review board and received a waiver.

### Beth Israel Deaconess Medical Center (MIMIC-IV)

The BIDMC data were accessed as de-identified data from The Multiparameter Intelligent Monitoring in Intensive Care (MIMIC-IV) v0.1 database released on 15 August 2019. MIMIC-IV is an update to the MIMIC-III single center public dataset made available to members of the Massachusetts Institute of Technology Laboratory for Computational Physiology Clinical Data Consortium prior to open public release.

- Reference: Johnson AEW, Pollard TJ, Shen L, et al. MIMIC-III, a freely accessible critical care database. Sci Data. 2016;3:160035. doi:10.1038/sdata.2016.35

Inclusion Flow Diagram
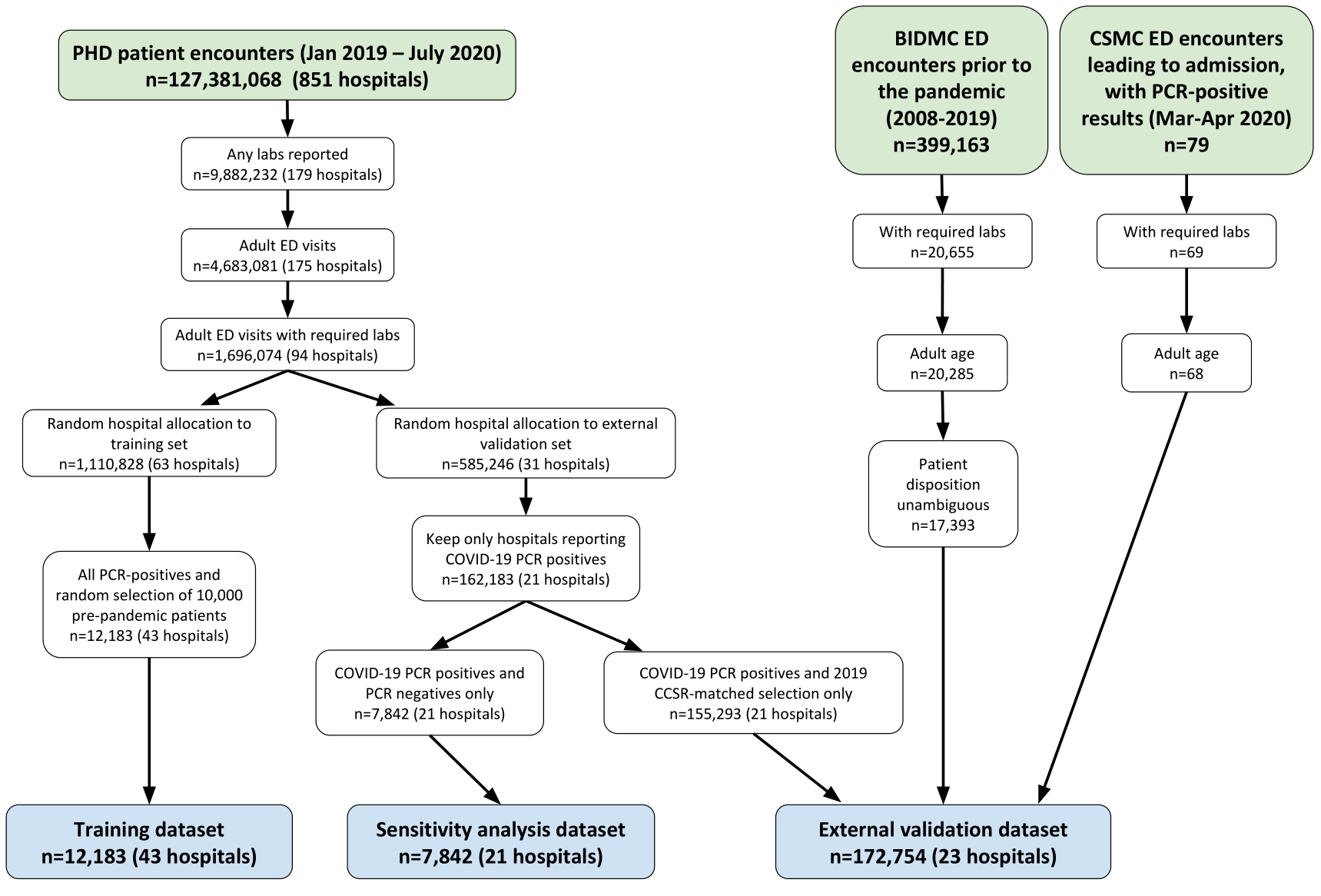


Abbreviations: BIDMC, Beth Israel Deaconess Medical Center; CCSR, Clinical Classifications Software Refined; CSMC, Cedar-Sinai Medical Center; ED, emergency department; MPHD, Premier Healthcare Database; PCR, reverse transcription polymerase chain reaction.

Additional details for PHD patients: The pre-pandemic window was Jan 2019-Dec 2019, and negative controls for the training and external validation datasets came from this timeframe. Cases in the training and external validation datasets were COVID-19 PCR positive patients from the Mar 2020-July 2020 timeframe. Patients who sought care in Jan or Feb 2020 were not included in the training or external validation datasets. The sensitivity analysis included COVID-19 PCR positive (cases) and PCR negative (negative controls) patients in the Mar 2020-July 2020 timeframe.

## Section B. List of CCSR Codes Used to Select Controls in the PHD Holdout Dataset

Clinical Classifications Software Refined (CCSR) are groupings of clinically-related International Classification of Diseases (ICD) codes and were obtained from: *Clinical Classifications Software Refined (CCSR) for ICD-10-CM Diagnoses. Healthcare Cost and Utilization Project (HCUP).* May 2020. Agency for Healthcare Research and Quality, Rockville, MD. www.hcup-us.ahrq.gov/toolssoftware/ccsr/ccs_refined.jsp.

- CIR008 - Hypertension with complications and secondary hypertension
- CIR012 - Nonspecific chest pain
- DIG017 - Biliary tract disease
- DIG022 - Noninfectious gastroenteritis
- END003 - Diabetes mellitus with complication
- END011 - Fluid and electrolyte disorders
- FAC016 - Exposure, encounters, screening or contact with infectious disease
- GEN004 - Urinary tract infections
- GEN005 - Calculus of urinary tract
- INF002 - Septicemia
- MBD017 - Alcohol-related disorders
- NVS010 - Headache; including migraine
- PRG028 - Other specified complications in pregnancy
- RSP002 - Pneumonia (except that caused by tuberculosis)
- SYM001 - Syncope
- SYM004 - Nausea and vomiting
- SYM006 - Abdominal pain and other digestive/abdomen signs and symptoms
- SYM012 - Circulatory signs and symptoms
- SYM013 - Respiratory signs and symptoms
- SYM015 - General sensation/perception signs and symptoms

## Section C: Feature Importance and AUROCs

Feature importance are defined as the values extracted in the *feature_importances_* property of the XGBoost model.

| Feature | Importance | AUROC |
| --- | --- | --- |
| Eosinophils (%) | 23.62 | 77.56 |
| Calcium total (mg/dL) | 16.85 | 75.60 |
| AST (IU/L) | 9.45 | 70.63 |
| WBC (K/uL) | 6.73 | 65.72 |
| Basophils (%) | 6.36 | 63.97 |
| RDW (%) | 5.13 | 55.03 |
| RBC (m/uL) | 5.11 | 58.05 |
| Albumin (g/dL) | 4.65 | 67.00 |
| Bilirubin total (mg/dL) | 4.30 | 51.46 |
| MCV (fL) | 3.98 | 59.36 |
| MCH (pg) | 3.49 | 57.34 |
| Sodium (mEq/L) | 3.29 | 62.98 |
| Bicarbonate (mEq/L) | 2.97 | 61.65 |
| BUN (mg/dL) | 2.40 | 55.42 |
| Chloride (mEq/L) | 1.68 | 56.53 |

Abbreviations: AST, aspartate aminotransferase; AUROC, receiver operating characteristic; BUN, blood urea nitrogen; MCH, mean corpuscular hemoglobin; MCV, mean corpuscular volume; RBC, red blood cell count; RDW, red cell distribution width; WBC, white blood cell count.

**Supplemental Table S1.** Summary of laboratory results grouped by panel for patients included in study – by COVID-19 status^1^.

| Laboratory Result, Median (Interquartile Range) | | Training Dataset | | Validation Dataset | | Sensitivity Dataset | |
| --- | --- | --- | --- | --- | --- | --- | --- |
|  |  | Negative | Positive | Negative | Positive | Negative | Positive |
| Basic metabolic panel | |  |  |  |  |  |  |
|  | Sodium (mEq/L) | 139.0 (4.0) | 137.0 (4.0) | 139.0 (4.0) | 137.0 (4.0) | 138.0 (4.0) | 137.0 (4.2) |
|  | Chloride (mEq/L) | 103.0 (5.0) | 102.0 (6.0) | 103.0 (6.0) | 102.0 (6.0) | 103.0 (6.0) | 102.0 (6.0) |
|  | Bicarbonate (mEq/L) | 25.0 (4.0) | 24.0 (4.0) | 24.5 (5.0) | 24.0 (5.0) | 24.0 (5.0) | 24.0 (4.1) |
|  | Blood urea nitrogen (mg/dL) | 14.0 (9.0) | 12.6 (11.0) | 14.0 (9.0) | 13.0 (10.0) | 15.0 (10.0) | 12.0 (10.0) |
|  | Calcium total (mg/dL) | 9.3 (0.7) | 8.7 (0.7) | 9.3 (0.7) | 8.6 (0.8) | 9.2 (0.7) | 8.6 (0.9) |
| Liver function panel | |  |  |  |  |  |  |
|  | Albumin (g/dL) | 3.9 (0.8) | 3.5 (0.8) | 4.0 (0.7) | 3.6 (0.8) | 3.9 (0.7) | 3.6 (0.7) |
|  | Aspartate Aminotransferase (IU/L) | 22.0 (14.0) | 34.0 (28.0) | 22.0 (14.0) | 33.0 (29.0) | 23.0 (17.0) | 32.0 (28.0) |
|  | Bilirubin total (mg/dL) | 0.5 (0.4) | 0.5 (0.3) | 0.5 (0.4) | 0.5 (0.4) | 0.5 (0.4) | 0.5 (0.4) |
| Complete blood count, differential | |  |  |  |  |  |  |
|  | White blood cell count (K/uL) | 8.2 (4.1) | 6.5 (3.7) | 8.4 (4.4) | 6.0 (3.5) | 8.9 (5.1) | 6.0 (3.5) |
|  | Red blood cell count (m/uL) | 4.5 (0.9) | 4.7 (0.8) | 4.5 (0.8) | 4.7 (0.9) | 4.5 (0.9) | 4.7 (0.8) |
|  | Mean corpuscular volume (fL) | 89.7 (7.4) | 87.7 (6.8) | 89.3 (7.2) | 87.7 (7.1) | 89.8 (7.9) | 87.7 (7.1) |
|  | Red blood cell Distribution width (%) | 13.5 (1.9) | 13.1 (1.6) | 13.2 (1.9) | 13.0 (1.5) | 13.5 (2.2) | 13.0 (1.5) |
|  | Mean corpuscular hemoglobin (pg) | 29.8 (2.9) | 29.2 (2.6) | 29.7 (2.9) | 29.1 (2.7) | 29.7 (3.0) | 29.1 (2.7) |
|  | Basophils (%) | 0.4 (0.7) | 0.2 (0.4) | 0.4 (0.5) | 0.2 (0.2) | 0.4 (0.6) | 0.2 (0.2) |
|  | Eosinophils (%) | 1.4 (2.0) | 0.1 (0.9) | 1.2 (1.9) | 0.1 (0.7) | 1.0 (1.9) | 0.1 (0.7) |

**^1^**Definitions for the training, validation, and sensitivity datasets appear in the footer for Table 1.

**Supplemental Figure S1.** Fifteen included laboratory features in the training dataset, by COVID‑19 status^1^.


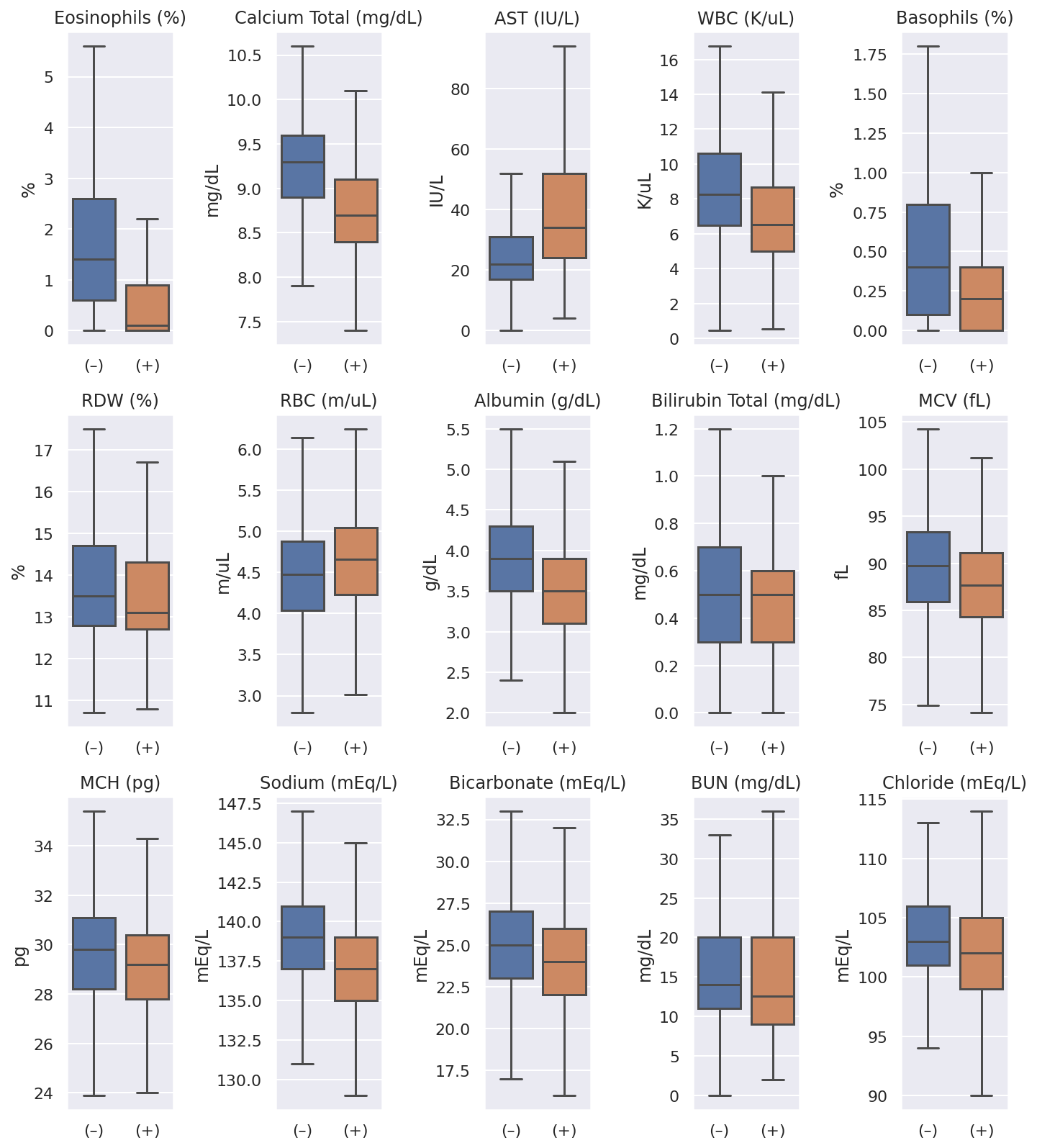


Abbreviations: AST, aspartate aminotransferase; BUN, blood urea nitrogen; MCH, mean corpuscular hemoglobin; MCV, mean corpuscular volume; RBC, red blood cell count; RDW, red cell distribution width; WBC, white blood cell count.

^1^Boxplots for all features in the training data set, ordered by feature importance to the machine learning model (see **Supplemental Appendix C**), stratified by COVID-19 negative (blue) and positive (orange) status.

**Supplemental Figure S2.** AUROCs for individual laboratory features and the overall model^1^.


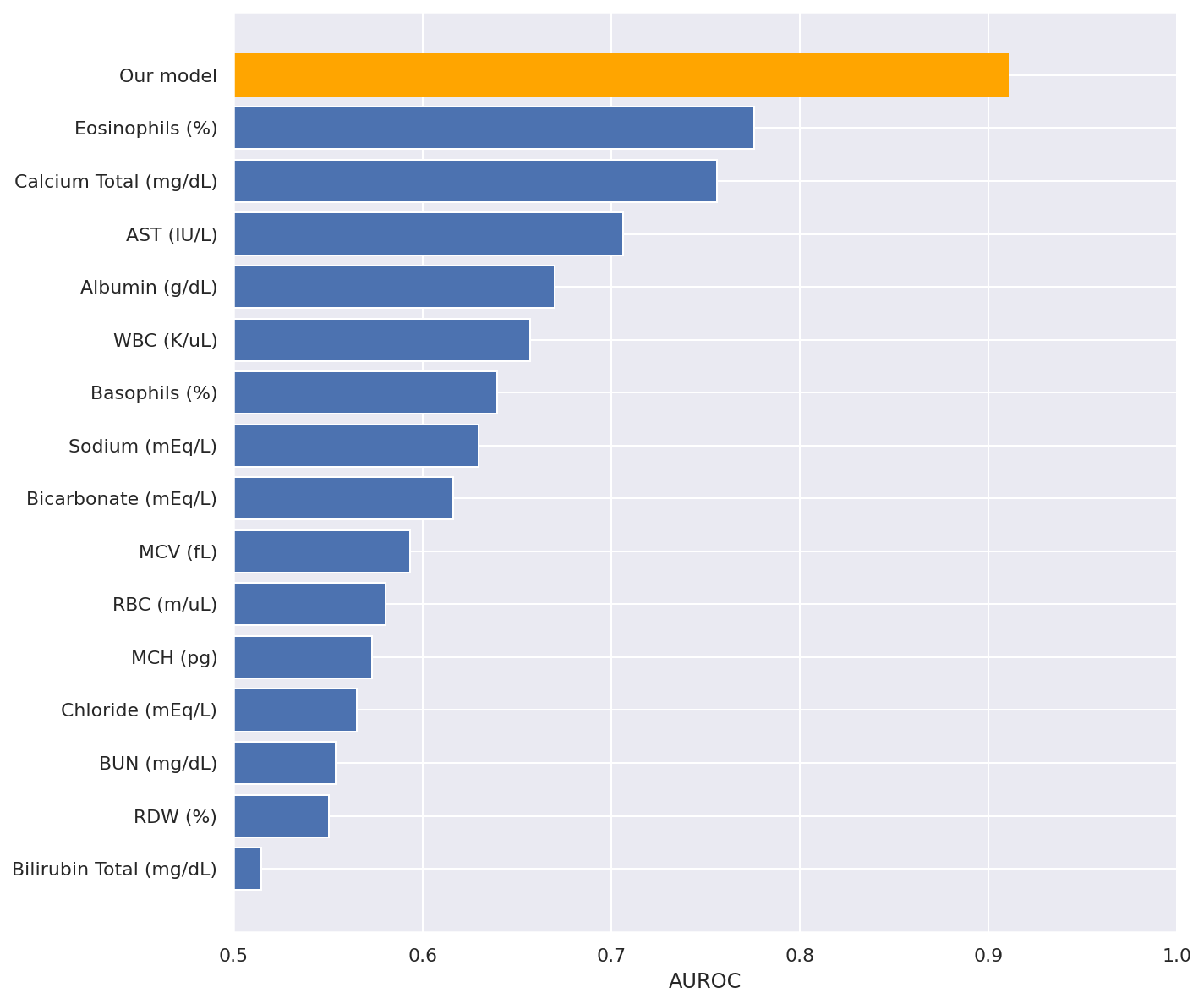


Abbreviations: AST, aspartate aminotransferase; AUROC, receiver operating characteristic; BUN, blood urea nitrogen; MCH, mean corpuscular hemoglobin; MCV, mean corpuscular volume; RBC, red blood cell count; RDW, red cell distribution width; WBC, white blood cell count.

^1^A list of all inputs used by the model and their AUROC during cross-validation in the training set, ordered by AUROC (note this ordering is distinct from the one in **Supplemental Figure S1**, which is ordered by importance to the machine-learning model). All these markers are laboratory tests present in either a complete metabolic panel or complete blood count. The individual markers with the best predictive power are eosinophils and total calcium, which by themselves have only fair performance (AUROC <0.80). However, by combining all the listed markers through our machine-learning model, a substantially higher AUROC is achieved (0.91).

**Supplemental Figure S3.** AUROC in demographic subgroups and by patient disposition in the sensitivity analysis dataset^1^.


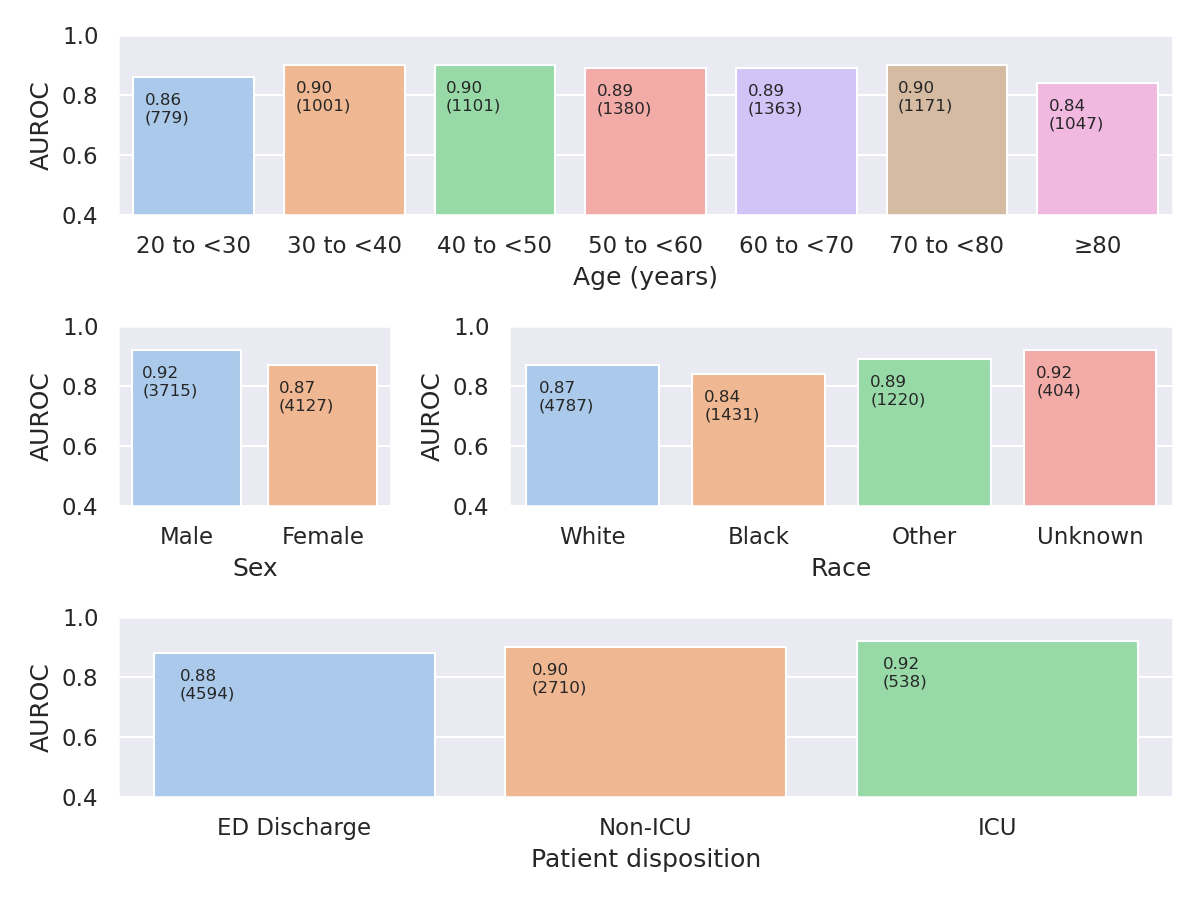


Top numbers are AUROC curves, bottom numbers in parentheses are the number of patients. AUROC: area under the receiver operating characteristic; ED: emergency department; ICU: intensive care unit.

**Supplemental Figure S4.** Distribution of risk scores in populations from PHD, DSMC, and BIDMC datasets^1^.

**
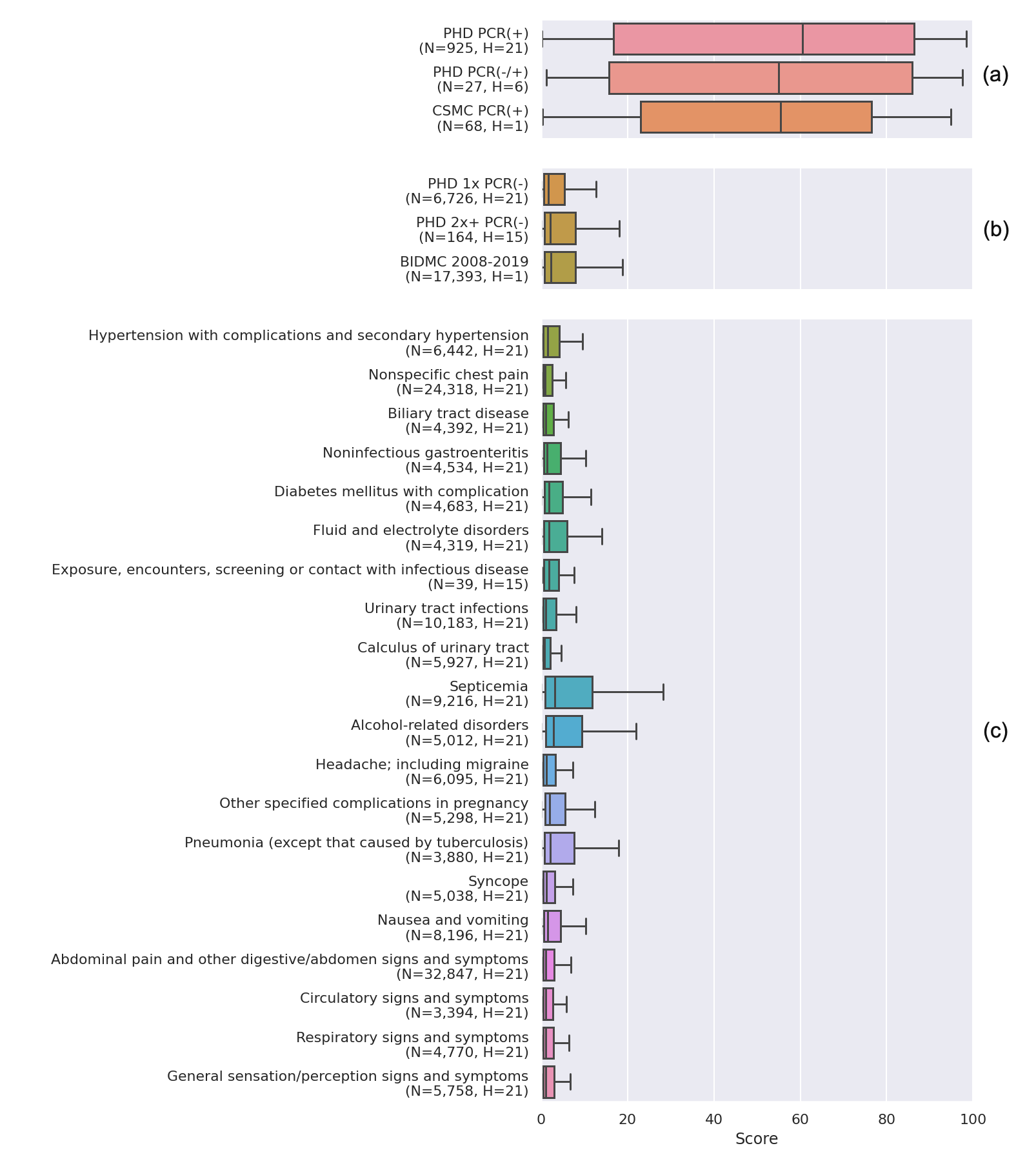
**

Abbreviations: BIDMC, Beth Israel Deaconess Medical Center; CCSR, Clinical Classifications Software Refined; CSMC, Cedar-Sinai Medical Center; PCR, reverse transcription polymerase chain reaction; PHD, Premier Healthcare Database.

**^1^**N is the number of patient visits, H is the number of hospitals in each cohort. Higher scores correspond to higher likelihood of a patient being COVID-19-positive. **(a)** PCR-confirmed positive cases, including those who first had a negative then a subsequent positive result on the same day, denoted “PCR (-/+)”. **(b)** PCR-negative patients, either with a single negative test result (1x) or with 2 or more negatives (2x+), as well as pre-pandemic patients from BIDMC. **(c)** 2019 patients from the PHD with primary diagnosis matching the top 20 non-COVID diagnoses in 2020, as grouped by CCSR diagnostic codes (cf. **Supplemental Appendix B**).
